# Virtual reality and travel anxiety during the COVID-19 pandemic: the moderating role of blockade intensity

**DOI:** 10.3389/fpsyg.2023.1287765

**Published:** 2024-08-30

**Authors:** Qingjin Wang, Renbo Shi, Kaiyun Zhang, Xiao Liu

**Affiliations:** ^1^Business School, Qingdao University, Qingdao, China; ^2^School of Economic and Management, Beihang University, Beijing, China

**Keywords:** virtual reality, travel anxiety, lockdown, virtual travel, COVID-19

## Abstract

The COVID-19 pandemic has deprived travelers of the right to continue their travel or leisure activities, while creating concerns about the safety of travel. In view of the great impact of the COVID-19 pandemic on travelers, we discussed the impact of virtual reality on travel anxiety during the COVID-19 pandemic, and considered the regulatory effect of blockade intensity. In order to explore the relationship between virtual reality and travel anxiety in depth, this study conducted a questionnaire survey on 299 Chinese tourists who had experienced virtual reality activities related to travel, and empirically analyzed the questionnaire data using SPSS 26 software. The results show that virtual reality has a significant negative effect on travel anxiety during the COVID-19 pandemic, i.e., virtual reality technology can provide a safer virtual travel experience for people and reduce their travel anxiety. At the same time, the relationship between virtual reality and travel anxiety varied to some extent depending on the intensity of the lockdown in each region, with the mitigating effect of virtual reality on travel anxiety being enhanced by high levels of lockdown. Therefore, we believe that although lockdown policies are necessary for some time to come, travel companies need to make further efforts to provide more convenient virtual reality services to alleviate travel anxiety caused by COVID-19 pandemic and lockdown to tourists. At the same time, virtual reality opens up new ideas for travel businesses under the impact of COVID-19 and contributes to the sustainable development of the travel industry.

## 1 Introduction

As of June 2020, more than 54 million people have been diagnosed with pneumonia caused by COVID-19 worldwide, resulting in more than 6.32 million deaths. Governments around the world have imposed restrictions such as border closures, community lockdowns, flight suspensions, and public place closures to avoid the spread of the epidemic (Yao et al., 2022). It is clear that all these restrictions fundamentally disrupt the global tourism market and its mobility (Gössling et al., 2020). According to the travel restrictions report published by the United Nations World Tourism Organization (UNWTO), travel restrictions related to the COVID-19 pandemic are implemented in 100% of global destinations (United Nations World Tourism Organization [UNWTO], 2020), while domestic tourism activities are prohibited by COVID-19 disease policies at the national level. Studies have shown that people experience unpleasant states when behavioral freedom is eliminated or threatened by uncontrolled events (Irimiás and Mitev, 2021b). This unexpected restriction of daily social and recreational activities deprives them of their rights as tourists, triggering a strong desire to expect a return to normalcy in tourism. In this situation, tourists and travelers are unable to continue traveling or engaging in leisure activities, they become fearful of risking their lives while traveling, become concerned about travel safety, and even become nervous or anxious about traveling when they see news about COVID-19.

One study found that because people are confined to their homes, a large group of people are shifting their activities to the virtual world, significantly increasing the frequency of virtual travel experiences using virtual reality (VR) technology (Leung et al., 2022). In the tourism industry, VR can be used to create virtual environments that enable virtual travel experiences by providing synthesized or realistically filmed 360-degree content, as well as powerful non-immersive, semi-immersive, or fully immersive VR systems that stimulate the visual and potentially other senses (Beck et al., 2019). VR-based travel allows travelers to comply with government-mandated social distance or lockdown requirements while still allowing for a valuable consumer experience (Itani and Hollebeek, 2021). Although VR was previously seen as a threat to travel, he today offers a safe travel opportunity for people to overcome the challenges of a pandemic, and virtual reality technology can provide a safer virtual travel experience that refreshes their mental state when they are anxious and concerned about the feasibility and safety of travel during a pandemic.

Furthermore, we believe that when travelers are completely restricted from traveling due to the potential risk of COVID-19 infection, it will be more difficult to inhibit their willingness to travel and will more likely motivate them to shift their interest from the unbalanced real world to the near-ideal virtual reality, thus alleviating realistic travel anxiety. In order to contain COVID-19 pandemic, most governments have imposed lockdowns, yet the extent of the lockdown depends on the severity of the COVID-19 pandemic, and people in areas with severe outbreaks are even strictly required to stay at home. Tourists in high lockdown situations have stronger cognitive desires and emotional experiences than those in areas with lower levels of lockdown (Irimiás and Mitev, 2021a). Psychological reactance theory (Miron and Brehm, 2006) suggests that individuals experience resistance when their behavioral freedom is threatened, restricted, or lost, and that psychological reactance motivates behavioral and emotional efforts to cope with the deprivation of their freedom. We therefore argue that tourists in highly lockdown areas are more willing to travel due to the severe restrictions on their freedom, and the more restricted they are, the more likely they are to choose to release their travel anxiety through virtual means, and the more profound the travel experience using virtual reality technology.

Based on the above discussion, previous studies have examined the role of VR in heritage preservation (Bec et al., 2019), retailing (Wedel et al., 2020), and consumer perception (Park and Yoo, 2020), and for tourism, scholars have focused on the impact of virtual travel on realistic travel intentions (Bogicevic et al., 2019), destination marketing (Lin et al., 2020), tourism recovery (El-Said and Aziz, 2022), tourist behavior intention (Leung et al., 2022), tourist sentiment (Zhang et al., 2022), etc., while ignoring the effect of virtual reality on travel anxiety during COVID-19. In fact, virtual reality technology is being used in daily life. Whether it can reduce passengers’ travel anxiety, e.g., by providing virtual experience, training, reassurance and self-control, has not been answered by existing studies. By surveying 299 Chinese respondents on their perceptions of VR and travel during the COVID-19, our study applies the theory of psychological reactance to explore the impact of virtual reality anxiety during the COVID-19 and validates the moderating role of lockdown policies between the two. From a theoretical perspective, the study expands the contextual application of psychological resistance theory and explores the complex mechanisms of virtual reality’s impact on travel anxiety. From a practical perspective, the study provides practical guidance for travel companies on how to reduce travel anxiety during the COVID-19.

## 2 Literature review

### 2.1 Virtual tour application

As virtual reality technology enters and becomes a high-tech that attracts much attention in the field of tourism (Lin et al., 2020). Researchers have conducted rich studies based on the supply side perspective, exploring the application of virtual reality technology in scenic area development, destination services and marketing.

Virtual reality has helped tourist attractions to realize digital and 3D design. VR digital scenic design based on ArcGIS (Xu et al., 2009), 3D-GIS (Huang et al., 2016), and virtual reality design of scenic spots with 3D model technology (Poux et al., 2020) have been widely used. Many tourist attractions have developed game experiences based on virtual reality technology, such as Orava Castle in Czechoslovakia, where a cell phone AR game was developed (Mesáro et al., 2016), and Geevor Tin Coal Mining Site in England, where an AR treasure finding game was developed (Jung and Tom Dieck, 2017) to enhance the interactive experience of tourists. Virtual reality technology also plays an important role in destination services and marketing. For example, the smartphone application APP Mtrips applies AR to city travel guides, where users take a view by using the smartphone camera, and detailed information such as attraction ratings of tourist destinations will be displayed on the phone, serving as a service recommendation and word-of-mouth marketing.

### 2.2 Virtual travel experience

Virtual reality creates a new travel platform and travel mode for tourists, bringing them a new travel experience. Based on the content analysis of existing virtual tourism literature, this paper understands virtual tourism experience from two aspects: virtual tourism hardware experience and psychological experience.

Existing literature mainly focuses on user acceptance and perceived evaluation of virtual tours. Perceived usefulness and perceived ease of use have been commonly used by existing researchers to evaluate people’s acceptance of virtual tours. Chung and Koo (2015) proposed a model that includes a combination of technology readiness level, visual attractiveness, and convenience to reveal consumer acceptance of virtual tours. Javornik (2016) argued that the hardware responsiveness and control affects people’s acceptance of virtual tours. Tourists’ perceived evaluation of virtual tour technology involves factors such as functionality, content, technical ease of use, and interactivity. Olsson et al. (2013) revealed four major dimensions of virtual tour functionality, including information accessibility, navigability, interactivity, and novelty.

Virtual reality technology is characterized by visualization, immersion and interactivity, which can quickly reproduce real tourist attractions and bring a new revolution to the tourism experience. Existing research on virtual tourism experience mainly focuses on sensory enjoyment and emotional experience, and sense of presence and sense of immersion are often used to measure the sensory enjoyment of virtual tourism users. Sense of immersion refers to the degree of immersion of users in virtual reality, and sense of presence refers to the user’s feeling of being present in the virtual environment. Jung and Tom Dieck (2017) revealed the positive effects of social presence on tourists’ educational experience, aesthetic experience, entertainment experience, and escape experience in a museum’s virtual reality scenario. Hyun and O’Keefe (2012) found that tourist information helps to construct a sense of presence in the virtual context, and that the sense of presence facilitates the formation of virtual cognitive-emotional imagery of the destination. Spielmann and Mantonakis (2018) identified the sense of interaction of human-computer interaction in the virtual tourism experience as an important antecedent factor affecting the sense of presence.

### 2.3 Travel intention

Some scholars have explored the antecedent mechanisms that influence tourism willingness and behavior from different perspectives. For example, Shoukat et al. (2023a) investigated the antecedents of nostalgia related cultural travel behavior. They found that tourists’ intrinsic, integrated, and identified motivations help to awaken their willingness to revisit, which in turn has a positive impact on actual visiting behavior. The willingness to revisit plays a strong mediating role between actual visiting behavior and autonomous motivation. In addition, they also found that the image of the visited destination and the past experiences of tourists can affect the willingness to revisit, thereby having a positive impact on actual visiting behavior. Building on previous research, Shoukat et al. (2023b) investigated the travel willingness of medical tourists in the post pandemic era. They found that the Medical Travel Index helps alleviate travel anxiety and fear among tourists, thereby increasing their willingness to travel to countries with unique medical tourism potential. Travel anxiety mediates the correlation between medical travel index and travel intention. In addition, they also found that the perceived severity of the COVID-19 epidemic will increase the travel anxiety of medical tourists.

### 2.4 Virtual tourism and sustainable tourism development

More researchers believe that virtual tourism has a positive impact on the sustainable development of tourism, which is mainly reflected in the following two aspects. First, virtual reality technology as a new tool for tourism marketing, consumers can use the technology for pre-trip pre-experience before making travel decisions (Lin et al., 2020). Virtual reality technology provides a rich environmental resource for potential tourists to pre-explore destinations, allowing tourists to experience destinations in a holistic and immersive way before traveling (Huang et al., 2016). Pantano and Servidio (2011) found that virtual tour participants aspired to real tourist attractions and compared real attractions to virtual ones. As consumers’ virtual tour participation increased, their positive feelings toward the destination increased (Kim et al., 2020), which positively influenced their attitudes toward travel, and travel behavior decisions (Lin et al., 2020). Marasco et al. (2018) found that the emotional experience of virtual tours based on wearable devices positively affects the willingness to visit cultural heritage sites. Secondly, virtual tours are considered as a complementary approach to field tourism experiences. The combination of virtual reality and real environment, through augmented reality (AR), mixed reality (MR), and other virtual reality technologies, can enrich the dimensions of the field tourism experience and enhance tourists’ sense of reality experience (Guttentag, 2010).

## 3 Research hypotheses

### 3.1 Virtual reality and travel anxiety

Virtual Reality (VR) is a computer-generated scenario used to simulate immersive, life-like experiences based on reality. Virtual reality technology offers a new way of traveling that can help consumers overcome the geographical constraints of travel and enjoy a realistic travel experience at the attractions they want to travel to Subawa et al. (2021). Virtual reality can be realized by creating a virtual environment that provides synthetic or 360-degree realistically captured content and a powerful non-immersive, semi-immersive, or fully immersive VR system to achieve a virtual travel experience that stimulates the visual and potentially other senses.

Virtual travel technology can increase consumer engagement, provide them with a safer virtual travel experience, and alleviate travel anxiety due to COVID-19 blockade within. Attractions (e.g., museums, theme parks) had already begun to adopt virtual reality technology for enhanced user experiences prior to the outbreak of COVID-19 (Li et al., 2021). During the period of COVID-19, more and more travel companies introduced virtual reality services to provide travelers with a virtual travel experience, and virtual reality technology has become an important platform for travel companies to maintain revenue (Merkx and Nawijn, 2021). Due to the potential contagiousness of COVID-19, governments have implemented embargo policies to varying degrees, and tourists who have their freedom restricted are like birds in a cage; according to the theory of psychological resistance, the more tourists are restricted, the stronger their desire to travel (Li et al., 2021). At the same time, due to the health threat of COVID-19, tourists also have concerns about travel safety, and thus may experience varying degrees of travel anxiety (Walters et al., 2022). Virtual Reality (VR) can help travelers to travel virtually by using computer-generated images or videos that simulate real-life experiences and provide safe travel options during COVID-19. Travelers will feel a sense of presence in a highly immersive virtual environment, which will reduce stress and worry about the safety and feasibility of travel, and alleviate internal travel anxiety. In summary, the following hypotheses are proposed:

**H1:** Virtual reality can alleviate travel anxiety and has a negative effect on travel anxiety.

### 3.2 Moderating effects of blockade intensity

When tourists are completely restricted from traveling due to the potential risk of COVID-19 infection, it will be more difficult to suppress their inner willingness to travel, and it is more likely to motivate them to shift their interest from the unbalanced real world to the near-ideal virtual reality, thus alleviating the reality of travel anxiety (Iacovino et al., 2020). In order to contain COVID-19, most governments imposed a blockade, yet the extent of the blockade depended on the severity of the COVID-19 pandemic, with people in areas with severe outbreaks even being severely restricted from traveling. Tourists under high levels of blockade develop stronger cognitive desires and emotional experiences compared to those in areas with lower levels of blockade (Li et al., 2021). Psychological resistance theory suggests that when an individual’s freedom of behavior is threatened, restricted, or lost, they resist the restriction, prompting behavioral and emotional efforts to cope with the deprivation of their freedom (Akhtar et al., 2021). Therefore, tourists in highly blocked areas have a stronger willingness to travel due to severe restrictions on their freedom, and the higher the degree of restriction, the more likely tourists are to choose virtual reality as a way to release their inner travel anxiety, and the travel experience they get by using virtual reality technology will be more profound. In summary, the following hypotheses are proposed:

**H2:** Blocking intensity has a positive moderating effect in the process of virtual reality affecting travel anxiety.

## 4 Materials and methods

### 4.1 Questionnaire design

According to the standardized questionnaire design process, the questionnaire process used in this study is as follows:

First, determine the research variables and construct a theoretical model. On the basis of literature combing on virtual reality, travel anxiety, and blockade intensity, this paper further analyzes the role relationship between virtual reality, travel anxiety, and blockade intensity, and constructs a theoretical research model.

Second, the design of the initial scale. Focusing on the related studies of virtual reality, travel anxiety, and blockade intensity, the domestic and international mature scales that are most compatible with the research background and content of this paper, with high citation rate, qualified reliability and validity, and published in international Top journals are collated and screened out, and selected as the initial scales of this paper.

Third, questionnaire content design. The questionnaire content of this paper mainly contains two parts. (1) Description of the questionnaire. This part describes the research purpose of the questionnaire, the main content involved and the rules for filling in the questionnaire, etc., and in the questionnaire instructions, it is stated that “the information of the respondents will be kept strictly confidential to ensure that the results of the survey will only be used for academic research.” (2) The main part. This part contains the main variables involved in this paper (virtual reality, travel anxiety, blockade intensity) and the measurement of the control variables, all of which are closed-ended questions, and are mainly scored on a seven-point Likert scale.

Fourth, formal research. We randomly sampled 500 Chinese tourists who had experienced travel related virtual reality activities. A total of 326 questionnaire data were collected. After excluding three or more missing questions and those with obvious filling methods, a total of 299 valid questionnaires were obtained, with an effective response rate of 59.8%.

### 4.2 Sample and data collection

The purpose of this study was to examine the relationship between virtual reality and travel anxiety during COVID-19 pandemic and its relationship with lockdown intensity. This study was conducted in April 2022, when China was experiencing a new COVID-19 pandemic centered in Shanghai. Full lockdowns began in some areas and most domestic and international travel remained required to be closed. An online questionnaire was sent to 500 Chinese travelers who experienced travel-related virtual reality activities through a purposive sampling method. A total of 500 questionnaires were sent out by our team from April 2022 to June 2022, and 326 questionnaire data were collected. A total of 299 valid questionnaires were finally obtained by excluding those with 3 or more missing questions and those with obvious patterns of filling out the questionnaire, with a valid return rate of 59.8%.

### 4.3 Variable measurement

The dependent variable in this paper was travel anxiety during the COVID-19 pandemic, which was measured mainly by the measure developed and validated by Zenker et al. (2021) on a scale of 1 (strongly disagree) to 7 (strongly agree), and consisted of five measurement items, “I intend to try a virtual reality tour offered by an attraction’s Web site,” “I predict that I will use virtual reality services offered by an attraction’s Web site,” “I plan to visit an attraction using a virtual reality service,” “I am very likely to use a virtual reality service to visit an attraction,” and “I have already used virtual reality services offered by an attraction.”

The independent variable in this paper is virtual reality. The measure of virtual reality was mainly based on the measure developed and validated by Itani and Hollebeek (2021), with a scale ranging from 1 (strongly disagree) to 7 (strongly agree) and included 5 measures such as “COVID-19 makes me very worried about my normal way of traveling,” “Because of COVID-19, I am afraid of risking my life while traveling,” “When planning a vacation, the thought of C. neoformans pneumonia makes me uncomfortable,” “When I read news about C. neoformans pneumonia, I get nervous or anxious about traveling,” “I feel nervous or anxious about traveling,” and “I don’t feel safe to travel because of pneumococcal pneumonia.”

The moderating variable in this paper is lockdown. The measure of lockdown intensity was based on the measure developed and validated by Hale et al. (2021), with a scale ranging from 1 (strongly disagree) to 7 (strongly agree), including “closure of public transportation,” “cancellation of public events’,” “restricting international travel,” “schools switching to online instruction,” and “closure of public places (scenic spots, theaters, gyms, etc.),” “Limit gatherings (family and friend reunions, annual company meetings, etc.),” and “Home segregation” 7 measurement question items.

In addition, to investigate the relationship between the key explanatory variables and the explanatory variables, and to reduce the errors caused by individual differences in the regression results, we included age, education level, monthly income, whether one is a pandemic prevention and control worker, and whether one is confirmed as control variables in the model.

### 4.4 Equations

First, the first equation is used to test the effect of virtual reality on travel anxiety by creating the following OLS model for baseline regression:

$$Travel\;Anxiety_{i}=a_{0}+a_{1}\;Virtual\;Reality_{i}+\sum Control_{i}+{\text{γ}}_{i}$$

Where *TravelAnxiety*_*i*_ stands for travel anxiety, *VirtualReality*_*i*_ stands for Virtual Reality, i is individual, *Control*_*i*_ represents the control variable, γ_*i*_ is the standard error term.

Second, we added an interaction term between virtual reality and blockade intensity to the model to test the moderating effect of blockade intensity in the process of virtual reality influencing travel anxiety.

$$Travel\;Anxiety_{i}=c_{0}+c_{1}\;Virtual\;Reality_{i}+c_{2}\;Virtual\;Reality_{i}=$$

$$Blocking\;Intensity_{i}+\sum Control_{i}+{\text{δ}}_{i}$$

Where *TravelAnxiety*_*i*_ stands for travel anxiety, *VirtualReality*_*i*_ stands for Virtual Reality, i is individual, *Control*_*i*_ represents the control variable, γ_*i*_ is the standard error term.

## 5 Results

### 5.1 Common method variance tests

The data generated by the single respondent survey may have the problem of common method variance (CMV). In order to solve this problem, this study stated in the questionnaire guidelines that “respondents’ information will be kept strictly confidential and the results of the survey will be guaranteed to be used only for academic research” to prevent the accuracy of the survey results from being affected by the respondents’ intentional exaggeration of facts. In addition, this paper also evaluated the common method variance problem (CMV) through factor analysis with the Harman one-way ANOVA test. The results showed that the variance explained by the first principal component was 37.5% and the total variance explained was 74.6%, which did not exceed 50% of the total explained variance, indicating that the impact of the common method variance problem in this study was small.

### 5.2 Exploratory factor analysis

In this study, KMO and Bartlett’s test were conducted to test the appropriateness of the collected sample data. If the KMO is greater than 0.6, the validity of the scale is good; if the significance of the Bartlett’s test is less than 0.01, the scale of this study is qualified for factor analysis. The KMO and Bartlett’s test showed that the KMO value was 0.718. At the same time, the significance of Bartlett’s test is lower than 0.01, indicating that the study scale is suitable for factor analysis.

### 5.3 Reliability analysis

For the reliability test, we evaluated by calculating the Cranbach’s alpha coefficient and the combined reliability (CR). As shown in Table 1, the Cranbach’s alpha coefficient for virtual reality was 0.906, the Cranbach’s alpha coefficient for travel anxiety was 0.929, and the Cranbach’s alpha coefficient for blockade intensity was 0.937. Next, the factor loadings were calculated for each of the combined reliability (CR) corresponding to the variables. As shown in Table 1, the CR value of virtual reality was 0.926, the CR value of travel anxiety was 0.929, and the CR value of blockade intensity was 0.918. In summary, the Cranbach’s α coefficient values and CR values of all variables were above 0.7, indicating that the scale as a whole had good reliability. The results are shown in Table 1.

**TABLE 1:** Measurement index and results of reliability and validity testing.

Variable	Measurement index	References	Factor loadings	Cronbach’s alpha	CR	AVE
Virtual reality	I’m going to try the virtual reality tours offered by the attractions website.	Itani and Hollebeek, 2021	0.742	0.906	0.926	0.642
	I predict that I will use the virtual reality services offered by the attractions website.		0.736			
	I plan to use virtual reality services to visit attractions.		0.869			
	I will most likely use virtual reality services to visit attractions.		0.708			
	I have used the virtual reality services offered by the attraction.		0.778			
Travel anxiety	COVID-19 makes me very worried about my normal way of traveling.	Zenker et al., 2021	0.873	0.929	0.933	0.636
	Because of COVID-19, I am afraid to risk my life while traveling.		0.749			
	While planning my vacation, I felt sick thinking about the COVID-19.		0.746			
	I get nervous or anxious about traveling when I read the news about the COVID-19.		0.718			
	Due to the COVID-19, I feel unsafe to travel.		0.806			
Lockdown	Close public transportation	Hale et al., 2021	0.822	0.937	0.918	0.648
	Cancellation of public events		0.783			
	Restrictions on international travel		0.835			
	Schools switch to online teaching		0.716			
	Public places closed (scenic spots, theaters, gyms, etc.)		0.827			
	Restricted gatherings (family and friend reunions, annual company meetings, etc.)		0.736			
	Home Isolation		0.801			

### 5.4 Validity analysis

To test the validity of our constructed model, validated factor analysis (CFA) was first conducted to test the discriminant validity. The results of the analysis are shown in Table 2, and the three-factor model (χ^2^/ Df = 1.720, *p* < 0.05; RMSEA = 0.049, GFI = 0.930, CFI = 0.990, TLI = 0.988, IFI = 0.990) had the best fit indices compared to the two-factor and one-factor models. In addition, the square root of the AVE values of each variable is greater than the correlation coefficients with other variables, indicating that the study model has good discriminant validity among the constructs. In terms of convergent validity, the three-factor model fit was better (χ^2^/ Df = 1.720, *p* < 0.05; RMSEA = 0.049, GFI = 0.930, CFI = 0.990, TLI = 0.988, IFI = 0.990). Also, the factor loadings corresponding to the question items were all greater than 0.7 and the AVE values were all greater than 0.5, indicating that our measures had good convergent validity. The results are shown in Table 2.

**TABLE 2:** Confirmatory factor analysis results.

Model	*χ 2/Df*	RMSEA	GFI	CFI	TLI	IFI
Three-factor model	1.720	0.049	0.930	0.990	0.988	0.990
Two-factor model	16.846	0.231	0.556	0.775	0.741	0.776
Single-factor model	31.297	0.319	0.373	0.566	0.505	0.567

*N* = 299. Three factor model: virtual reality, travel anxiety, blockade intensity; Two factor model: merging virtual reality and travel anxiety, blockade intensity; Single factor model: All variables are merged.

### 5.5 Correlation analysis

To ensure that multicollinearity did not influence the results, descriptive statistical analyses were performed on the variables. The results showed that the correlation coefficients between all variables were below 0.70, indicating that the problem of multicollinearity is small. Also, as can be seen in Table 3, there was a significant negative correlation between virtual reality and travel anxiety (*r* = −0.590; *p* < 0.01), which is consistent with our research hypothesis. The specific results are shown in Table 3.

**TABLE 3:** Descriptive statistics and correlation analysis.

Variables	Mean	S.D.	(1)	(2)	(3)	(4)	(5)	(6)	(7)	(8)
(1) Age	2.330	1.034	1.000							
(2) Education	4.330	1.165	-0.099	1.000						
(3) Income	2.500	0.748	0.079	0.0780	1.000					
(4) Worker	1.830	0.374	-0.263**	0.214**	-0.157**	1.000				
(5) Confirmed	3.760	0.626	-0.228**	0.203**	-0.058	0.372**	1.000			
(6) VR	5.020	1.572	0.062	-0.030	0.089	-0.057	0.086	1.000		
(7) Lockdown	4.930	1.669	0.065	-0.062	0.083	-0.051	0.053	0.644**	1.000	
(8) Travel anxiety	3.480	1.546	0.033	0.113	-0.085	0.101	-0.008	-0.590**	-0.593**	1.000

Standard errors are in parentheses; *N* = 299; **p < 0.01.

### 4.6 Regression analysis

In Table 4, we used OLS regression to examine the relationship between virtual reality and travel anxiety.

**TABLE 4:** Analysis of hypothesis test results.

Variables	Travel anxiety				
	M_1_	M_2_	M_3_	M_4_	M_5_
**Control variables**					
Age	0.064	0.102*	0.064	0.110*	0.103*
Education	0.117	0.091	0.117	0.074	0.073
Income	-0.087	-0.037	-0.087	-0.027	-0.027
Worker	0.101	0.060	0.101	0.060	0.059
Confirmed	-0.060	0.023	-0.060	0.028	0.027
**Independent variable**					
VR		-0.589**		-0.359**	-0.314**
**Moderating variable**					
Lockdown				-0.361**	-0.321**
**interaction term**					
VR*Lockdown					0.116*
*R* ^2^	0.033	0.370	0.033	0.446	0.454
Δ*R*^2^	0.033	0.337	0.033	0.413	0.008
F	2.023	28.617**	2.023	33.487**	30.104**

Standard errors are in parentheses; *N* = 299; **p < 0.01, *p < 0.05.

First, Model 1 and Model 2 examined the effect of virtual reality on travel anxiety. Model 1 contains the results of the baseline model with control variables only. In Model 2, we included the independent variable (Virtual Reality), and the results showed a significant negative effect of virtual reality on travel anxiety (β = −0.589, *p* < 0.01). As expected, virtual reality technology can provide a safer virtual travel experience for people and reduce their travel anxiety. Hypothesis 1 was tested.

Second, models 3, 4, and 5 tested the moderating effect of lockdown intensity in the process of virtual reality affecting travel anxiety. In model 5, we added the interaction term of virtual reality and lockdown intensity. As Table 4 shows, the coefficient of the interaction term between virtual reality and lockdown intensity was positive and statistically significant (β = 0.116, *p* < 0.05), indicating that lockdown intensity has a positive moderating effect in the process of virtual reality affecting travel anxiety. Hypothesis 2 was tested.

### 5.7 Robustness test

Due to the small number of questionnaires collected, there may be bias in the empirical results of this article. Therefore, this article further validates the main effect and the moderating effect of blocking intensity using the Process macro program. From Equation 1 in Table 5, it can be seen that the value of virtual reality is β is −0.5792, the *t*-value is −12.4991, and the confidence interval at the 95% level is (−0.6703, −0.4880), which does not include 0, indicating that virtual reality has alleviated the travel anxiety of tourists during the COVID-19 epidemic, and H1 has been verified again. In addition, from Equation 2, it can be seen that the β-value of the interaction terms normalized for virtual reality and blockade intensity is 0.0525, and the *t*-value is 2.0015; At the same time, the confidence interval at the 95% level is (0.0009, 0.1042), without including 0, indicating that blockade intensity has a moderating effect in the impact of virtual reality on travel anxiety. H2 has been once again verified.

**TABLE 5:** Robustness test results.

	Model 1					Model 2				
	β	SE	T	LLCI	ULCI	β	SE	T	LLCI	ULCI
**Control variables**										
Age	0.1525	0.0730	2.0899*	0.0089	0.2961	0.1545	0.0684	2.25908	0.0199	0.2892
Education	0.1205	0.0642	1.8772	-0.0058	0.2468	0.0962	0.0601	1.6011	-0.0221	0.2145
Income	-0.0766	0.0983	-0.7793	-0.2700	0.1168	-0.0555	0.0919	-0.6035	-0.2364	0.1254
Worker	0.2484	0.2162	1.1489	-0.1771	0.6740	0.2457	0.2021	1.2159	-0.1520	0.6435
Confirmed	0.0573	0.1271	0.4507	-0.1929	0.3075	0.0671	0.1188	0.5647	-0.1668	0.3010
**Independent variable**										
VR	-0.5792	0.0463	-12.4991**	-0.6703	-0.4880	-0.5665	0.1205	-4.7018**	-0.8037	-0.3294
**Moderating variable**										
Lockdown						-0.5598	0.1244	-4.5012**	-0.8046	-0.3150
**interaction term**										
VR*Lockdown						0.0525	0.0263	2.0015*	0.0009	0.1042
*R* ^2^	0.3703					0.4537				
F	28.6166					30.1044				

Standard errors are in parentheses; *N* = 299; **p < 0.01, *p < 0.05.

## 6 Discussion and conclusion

This study developed a theoretical framework to explore the impact of virtual reality on travel. Our study found that VR not only provides a viable alternative to traditional travel during pandemics but also opens up new avenues for the sustainable development of the tourism industry under the impact of COVID-19. To be more specific, the study found that VR can significantly alleviate travel anxiety, especially under high lockdown conditions, as it allows tourists to experience travel virtually, reducing concerns about travel safety and feasibility. This effect is enhanced by the intensity of lockdowns, with stronger travel desires arising under more restrictive conditions.

### 6.1 Theoretical contributions

Previous research has explored VR’s role in areas like marketing, attitude, and visitor in tourism (Tussyadiah et al., 2018; Lo and Cheng, 2020; Yung et al., 2021). However, the specific focus on VR’s impact on travel anxiety during COVID-19 is a novel aspect of the field. Furthermore, prior studies have looked at VR’s influence on realistic travel intentions, destination marketing, and tourist behavior (Li and Chen, 2019; Ying et al., 2022; Saleh, 2023; Wang et al., 2023), but this research uniquely integrates the concept of lockdown intensity as a moderating factor. Our study fills a gap in existing literature by linking VR technology with psychological aspects of travel during a pandemic, offering practical insights for the tourism industry during challenging times.

### 6.2 Practical implications

From a practical and managerial perspective, our study can provide some suggestions. Based on the significant positive impact of virtual reality on traveler travel anxiety during the COVID-19 pandemic, travel companies can take advantage of this virtual travel trend by preparing to change their business models and strategies. Traditional travel businesses can partner with technology companies to offer self-service virtual reality technology services to meet the growing demand for VR tourism from tourists during the COVID-19 pandemic. In addition, since VR tourism allows tourists to escape reality and reduce negative emotions, virtual reality business operators should be able to enhance the immersive experience in a virtual reality environment by creating role-playing stories or developing mini games to create a higher sense of immersion and presence for tourists. At the same time, virtual reality opens up new ideas for travel businesses under the impact of COVID-19 and contributes to the sustainable development of the tourism industry.

### 6.3 Implications and limitations

As with any study, there are limitations to our work. One notable limitation is the study’s geographical focus on China, which may limit the generalizability of the findings to other cultural contexts. In terms of future research directions, the article suggests exploring the impact of VR in different cultural contexts to understand how cultural differences might affect its effectiveness in reducing travel anxiety. Future research could expand to different cultural contexts to validate the findings and employ more objective measures of anxiety. Furthermore, the research primarily used self-reported measures, which could be subject to biases like social desirability or recall bias. Additionally, longitudinal studies could offer insights into the long-term effects of VR on travel anxiety. The study also opens avenues for exploring the impact of different types of VR experiences (e.g., interactive vs. non-interactive) on travel anxiety.

## Data availability statement

The original contributions presented in the study are included in the article/supplementary material, further inquiries can be directed to the corresponding authors.

## Ethics statement

Ethical review and approval was not required for the study on human participants in accordance with the local legislation and institutional requirements. Written informed consent from the patients/participants or patients/participants legal guardian/next of kin was not required to participate in this study in accordance with the national legislation and the institutional requirements.

## Author contributions

QW: Conceptualization, Writing – review and editing. RS: Investigation, Writing – review and editing. KZ: Writing – original draft. XL: Data curation, Methodology, Writing – original draft.
